# Critical Review on the Utilization of Handheld and Portable Raman Spectrometry in Meat Science

**DOI:** 10.3390/foods8020049

**Published:** 2019-02-01

**Authors:** Anel Beganović, Luzia Maria Hawthorne, Katrin Bach, Christian W. Huck

**Affiliations:** 1 Institute of Analytical Chemistry and Radiochemistry, CCB-Center for Chemistry and Biomedicine, Innrain 80/82, 6020 Innsbruck, Austria; anel.beganovic@uibk.ac.at; 2 Department of Food Technology and Nutrition, Management Center Innsbruck, Universitaetsstrasse 15, 6020 Innsbruck, Austria; Luzia.Hawthorne@mci.edu (L.M.H.); Katrin.Bach@mci.edu (K.B.)

**Keywords:** Raman spectroscopy, handheld, portable, mobile, meat, meat quality, review

## Abstract

Traditional methods for the determination of meat quality-relevant parameters are rather time-consuming and destructive, whereas spectroscopic methods offer fast and non-invasive measurements. This review critically deals with the application of handheld and portable Raman devices in the meat sector. Some published articles on this topic tend to convey the impression of unrestricted applicability of mentioned devices in this field of research. Furthermore, results are often subjected to over-optimistic interpretations without being underpinned by adequate test set validation. On the other hand, deviations in reference methods for meat quality assessment and the inhomogeneity of the meat matrix pose a challange to Raman spectroscopy and multivariate models. Nonetheless, handheld and portable Raman devices show considerable potential for some applications in the meat sector.

## 1. Introduction

Meat industry as well as meat science have put forth numerous analytical methods for meat quality assessment in order to ensure edibility, to be in compliance with control authority regulations and to offer consumers high quality products and satisfy their needs. Improvements of old and established technologies, technological advances [1,2] and the consumers’ constant demand for safe and guaranteed quality have made their contribution to this development. Apart from that, meat scandal headline news all over the world (e.g., horse meat scandal in Europe in 2013, export of rotten beef from Brazil in 2017 or reports on hygiene breaches in meat plants in the UK in 2018) raised consciousness about the quality of meat added to the growing interest and faster progress in meat quality assurance [3,4,5,6,7].

Among consumers and producers both, the taste experience and palatability of meat are considered to be the most important characteristics of meat and meat products. The palatability of meat is influenced by flavor, juiciness and tenderness, and according to Ref. [8] each of these three traits had a high correlation with the overall acceptability in a study with untrained consumer panels. Still, the evaluation of the mentioned meat quality parameters by human test persons is affected by subjective perceptions [9]. More objective tenderness assessment methods are for example the Warner-Bratzler shear force (WBSF) test [10] and particle size analysis based on the laser diffraction technique [11]. However, especially the popular and widely applied WBSF test shows discrepancies when compared to panel ratings—most likely due to the measurement setup and sample orientation [10,12]. A compact summary of instrumental methods for tenderness assessment can be found in Ref. [10].

Besides taste experience, the importance of the pH value, water-holding capacity (WHC, or drip loss) and L* value (lightness value) are well known parameters in meat science and play a central role in classifying meat into desired and undesired quality categories. As an example, reddish, firm and non-exudative (RFN) meat is considered as a desired pork meat quality by producers as well as consumers. On the other hand, reddish, soft and exudative (RSE); pale, soft and exudative (PSE); pale, firm and non-exudative (PFN) or dark, firm and dry (DFD) represent undesired pork meat quality categories. The categorization into one of these four categories of pork meat quality can be achieved by measuring the pH values at different times post mortem (i.e., 45 min and 24 h), the L* value and determining the drip loss over a defined period of time [13,14,15,16,17].

Intramuscular fat (IMF) and fatty acid (FA) composition are of great interest in the meat sector since they have a considerable impact on palatability and sensory characteristics of meat, and significantly contribute to the nutritional value. Furthermore, the European Union utilizes the lean meat content in order to classify pig carcasses into commercial categories (grades) [18]. As a result, IMF and FA need to be determined on a regular basis. A common method to quantify IMF in meat and meat products is the Soxhlet method according to the AOAC [19]. FA composition analysis is often conducted by extraction of FAs, followed by derivatization and purification steps, and completed by gas chromatography (GC) separation of FAs [20,21,22].

The detection of meat spoilage is of particular concern to producers as well as control authorities. The extent of microbial spoilage is usually determined as the number of colony-forming units (cfu) on plates with growth medium after a certain period of incubation [23,24]. With regards to reliable determination of the stage of meat spoilage, it seems rather difficult to establish competitive approaches to the method mentioned prior. Nevertheless, attempts of meat spoilage detection via the measurement of volatile organic compounds (VOCs) [25,26], or via applications utilizing electronic noses [27,28,29] can be found in literature.

Yet, the majority of all these described methods have obvious and non-negligible disadvantages. Most of them are invasive, meaning that the samples used for the determination of certain parameters are destroyed and thus cannot be used for resale. Another negative aspect of a variety of these methods is the necessity of time-consuming and complex sample pre-treatments, which often require trained and expensive personnel. Additionally, in case of tenderness determination, the recruitment of a (trained or untrained) sensory panel leads to subjective perceptions. All these factors contribute to a high financial expense for the producers, which are subsequently passed on to the consumers in the form of increased meat prices. Consequently, there is a demand for fast, non-invasive and cheap alternatives for meat quality assessment and assurance. Spectroscopic methods have the potential to fulfill these requirements, which is confirmed by numerous publications on this subject.

Near-infrared spectroscopy (NIRS) is a widely used technique for the characterization of meat and meat products in the meat sector [30,31,32,33]. Applications of other techniques in meat science, like attenuated total reflection mid-infrared spectroscopy (ATR-MIR) [34,35,36], visible-NIR spectroscopy (Vis-NIR) [37,38,39] or nuclear magnetic resonance (NMR) [40,41,42] are reported in literature as well. In the recent years there has been a growing interest in establishing Raman spectroscopy in this field of research [34,43,44], with reports on utilization of portable handheld Raman devices increasing especially. Thus, this review critically assesses the latest achievements and performances of portable handheld Raman spectrometers for the assessment of meat quality.

Raman spectroscopy is a non-invasive technique which is based on scattering of light on a molecular level. When photons collide with molecules, three different types of scattering occur: Rayleigh scattering, anti-Stokes Raman scattering and Stokes Raman scattering. If incident photons do not change their energy after colliding with molecules, the scattering is elastic and referred to as Rayleigh scattering. On the other hand, if incident photons do exchange energy with molecules, the resulting scattering is referred to as inelastic or Raman scattering. Raman scattering is caused by photons delivering energy to molecules (Stokes scattering) or receiving energy from molecules (anti-Stokes scattering). Due to this exchange of energy, transitions between energy levels in molecules are caused. Although Raman spectroscopy is focussed on vibrational energy level transitions, there are also transitions between electronic and rotational energy levels. Raman spectra can provide structural as well as qualitative information of a substance [45].

When it comes to the analysis of spectral data, multivariate methods are often used for the calibration of spectra with reference data. This calibration process is done with a calibration (or training) set and must contain all the variability which is expected in future samples. A modeling error called root mean square error of calibration (RMSEC) is obtained, which is often highly over-optimistic in terms of prediction ability. A closer estimate of a model’s future prediction performance of unknown samples is the root mean square error of cross validation (RMSECV). This error results from a cross validation procedure, where every sample is left out of the calibration once and subsequently predicted with the created calibration model. This approach is referred to as full cross validation or leave-one-out cross validation (LOO-CV). Another cross validation technique is know as segmented (or k-fold) cross validation, where a certain number of calibration samples are grouped into segments. Each segment is then left out of the calibration process once and predicted with the calibration model. Although the RMSECV represents a more realistic estimate of a model’s prediction ability then the RMSEC, the samples used in the cross validation process still originate from the calibration set. Thus, the cross validated error can not be considered as a reliable source of future prediction performance of a multivariate model on new and unknown samples. The only way to access an estimate of the expected error for the prediction of new and unknown samples is to use a set of samples with known reference values, which have never been involved in the calibration process. Such a set of samples is called test set or independent validation set and the resulting error is referred to as root mean square error of prediction (RMSEP). This validation step is essential in order to estimate the future model performance in predicting completely new and unknown samples and to avoid both overfitting and unterfitting. However, literature differs on the required size of the validation set—some sources suggest that the validation set should contain a similar number of samples as the calibration set, others recommend that approximately one third of the data should be kept out of the calibration in order to be used as test samples [46,47]. Due to the fact that meat is a very inhomogeneous sample matrix and subjected to a high level of biological variability due to various influences such as breed, feed, condition of livestock farming and age (to name just a few), a proper test set validation is mandatory for reliable statements on future samples. In our point of few, the ideal procedure for the development of spectroscopic applications in meat science would be to procure samples for the establishment of a calibration model at a time and after this, again procure new samples at another time for the purpose of testing the prediction ability (performance) of the developed calibration model. It is to be assumed, that other validation approaches than the one just mentioned above—especially cross validation—will lead to biased, less trustworthy or even useless and impractical multivariate models due to the already described reasons of pronounced inhomogeneity and biological variability of meat samples. Studies without an explicit independent validation set should point this out clearly in publications and draw conclusions carefully.

In the following chapters, a critical review of peer reviewed literature on the utilization of handheld and portable Raman spectrometers in meat science in the period of 2010 to 2018 will be presented. The advantages of handheld Raman devices in comparison to benchtop Raman spectrometers include the ability to perform in-field measurements (abattoir, retail, etc.), robust design as well as the usually simple handling. However, spectra of handheld devices are often less reproducible, less accurate and more affected by noise, which can be considered as a significant drawback—especially in food science and the therein faced complex matrices. For a comprehensive review on quality assessment of meat and fish with benchtop Raman devices, the interested reader is referred to Ref. [44].

## 2. Application of Raman Spectroscopy in Meat Quality Prediction

### 2.1. Prediction of Eating Quality Traits with Raman Spectroscopy

The sensory attributes of lamb and beef have been investigated with traditional methods and an attempt has been made to correlate these results with Raman spectra from a handheld device [Schmidt et al. (2013) [48], Fowler et al. (2014a, 2014b, 2015b, 2018) [49,50,51,52], Bauer et al. (2016) [53]]. One of the most frequently studied traits therein is tenderness using shear force measurements. All the attempts to correlate Raman spectra with shear force measurements can be considered as insufficient for routine application, as the best prediction error (root mean square error of prediction—RMSEP) using partial least squares regression (PLS-R) models was about 20% (normalized RMSEP—NRMSEP; see Equation (1)) of the calibration range with a coefficient of determination of $R_{\mathrm{VAL}}^{2}$ = 0.23 and 0.33 for two models with different data sets, respectively [Bauer et al. (2016) [53]]. The poor predictive power of shear force from Raman spectra with PLS-R can be elucidated with R^2^ values from Bauer et al. (2016) [53] using lamb samples. For one illustrative calibration model the authors obtain a very promising coefficient of determination of $R_{\mathrm{CAL}}^{2}$ = 0.94. The following cross validation and validation steps show a significant drop in the coefficient of determination to $R_{\mathrm{CV}}^{2}$ = 0.79 and $R_{\mathrm{VAL}}^{2}$ = 0.23.

(1) $$\mathrm{NRMSEP}=\frac{\mathrm{RMSEP}}{x{\left(\mathrm{CAL}\right)}_{\max}-x{\left(\mathrm{CAL}\right)}_{\min}}\times 100$$

Unfortunately, Bauer et al. (2016) [53] are the only ones that utilized an independent validation set (i.e., test set), although it is mandatory to test the performance of a PLS-R model [46] and despite the fact that the other publications had an adequate number of samples to at least test their PLS-R models with a few validation samples in predicting shear force of meat. Schmidt et al. (2013) [48] reported cross validation errors (RMSECV) between 26% and 31% for the prediction of shear force of 140 lamb samples from two different sites, but they did not mention how this percentage error was calculated. Independent of how this percentage error was calculated, the conclusion must be drawn very carefully whether the spectroscopic data from the Raman measurements can be correlated with shear force measurements as stated by the authors. Furthermore, they only reported $R_{\mathrm{CAL}}^{2}$ values, which can be considered as insufficient information when PLS-R has been used without any information on $R_{\mathrm{CV}}^{2}$ and $R_{\mathrm{VAL}}^{2}$. However, Fowler et al. (2014a, 2014b, 2015b, 2018) [49,50,51,52] concluded from their studies that there was little or no ability to predict shear force of lamb as well as beef using Raman spectra recorded with a handheld device. Figure 1 from Fowler et al. (2014) [49] clearly shows the poor correlation between reference and predicted shear force values. Table 1 gives a brief overview of the references which attempted to predict shear force and other target figures of various meat types using handheld Raman devices.

A more promising approach seems to be a classification of meat samples into tender and tough samples as described by Bauer et al. (2016) [53]. The authors set five different thresholds according to shear force measurements and investigated the ability of partial least squares discriminant analysis (PLS-DA) to differentiate between tender and tough beef samples from Raman spectra. The classification accuracy of the PLS-DA varied across the different thresholds between 59% and 80% correctly classified samples of the validation set (N = 75). Although the accuracy should be somewhat improved, this may find application in supporting producers to rapidly identify the toughest samples. However, it would have been interesting to see how other classification techniques would have performed, e.g., linear discriminant analysis (LDA) or support vector machine classification (SVM-C). Especially the performance of SVM-C would have been interesting since this method accounts for non-linear effects [54].

In the study of Fowler et al. (2018) [52] an untrained sensory panel was used to determine the sensory traits tenderness and juiciness of 45 beef loins in order to investigate potential correlations with Raman spectra. An error of RMSECV = 10.52 scores in a range of 19.8–82.6 scores is reported for predicting tenderness from Raman spectra measured with a handheld device and analyzed using PLS-R. According to Equation (1), this corresponds to an NRMSECV of approximately 17%, which might be acceptable, but it has to be mentioned that this error most probably will increase considerably when an independent test set is used for validation. Furthermore, there is a noticable drop in correlation from calibration to cross validation from $\rho_{\mathrm{CAL}}$ = 0.60 to $\rho_{\mathrm{CV}}$ = 0.47. The prediction of juiciness scores determined by the same sensory panel with Raman spectra yields an error of NRMSECV = 21% with PLS-R. It should be consequently concluded, and in contrast to the authors’ conclusion, that there is no sufficient ability of predicting juiciness scores with Raman spectroscopy. Although the authors continuously use the term RMSEP, they actually calculated an RMSECV. This can lead to misinterpretations of the reported errors (see Section 2.5, 3rd paragraph in Fowler et al. (2018) [52]).

### 2.2. Prediction of pH Values with Raman Spectroscopy

Since the pH is of particular importance to meat quality evaluation [63], serveral studies have tried to correlate pH measurements with Raman spectra obtained from handheld devices ready for mobile use [Scheier & Schmidt (2013) [55], Scheier et al. (2014, 2015) [56,57], Fowler et al. (2015b, 2018) [51,52], Nache et al. (2016) [58]]. In the study of Scheier & Schmidt (2013) [55] the pH values from 10 pork samples were recorded with a pH electrode as well as Raman spectra with a handheld device in a period from 30 min to 10 h post mortem. Subsequently, three different approaches were applied in order to predict the pH of pork from Raman spectra: peak intensity ratio of two Raman signals originating from phosphate groups at 980 and 1080 cm^−1^ (for more detailed information please refer to Scheier & Schmidt (2013) [55]), multiple linear regression (MLR) and PLS-R. Due to the lack of information on the pH range from 30 min to 10 h post mortem, the range was estimated herein using Figure 5 in Scheier & Schmidt (2013) [55] in order to get an idea of the error’s order of magnitude. As a result from this, the pH calibration range was determined to be approximately pH = 6.6–5.4. The peak ratio approach is reported to have a root mean square error of calibration (RMSEC) of 0.30 pH units with an $R_{\mathrm{CAL}}^{2}$ = 0.71. According to Equation (1), this yields an NRMSEC of 25%, which is rather high considering the fact that this is an error of calibration and no independent validation samples were used. The MLR and PLS-R approaches both show cross validation errors of RMSECV = 0.22 pH units and $R_{\mathrm{CV}}^{2}$ values of 0.70 (MLR) and 0.87 (PLS-R). With regard to the calibration range this error is roughly about NRMSECV = 18%. These values—especially the ones from the PLS-R—look somewhat promising, but it should be kept in mind that the sample set was quite small and that no validation set was used to test the performance of the cross validated models with independent samples.

**Figure 2:**
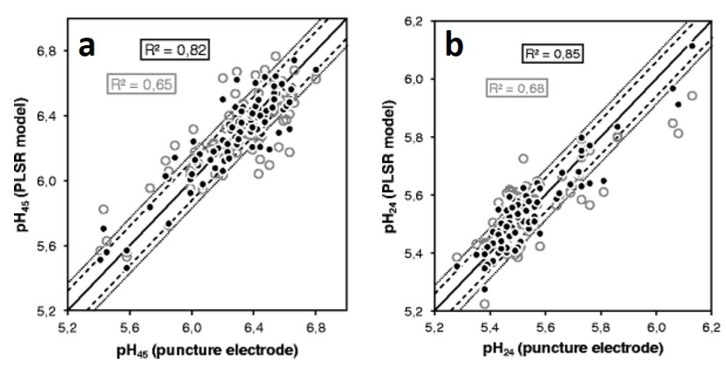
(**a**) Reference measurement of pH_45_ versus predicted pH_45_ from Raman spectra using PLS-R and cross validation. (**b**) Reference measurement of pH_24_ versus predicted pH_24_ from Raman spectra using PLS-R and cross validation. Calibration = black dots, cross validation = gray circles. Reprinted from Ref. [56] with permission from Springer Nature.

In the study of Scheier et al. (2014) [56] a larger sample set consisting of 96 pigs was used for pH measurements at 45 min (pH_45_) and 24 h (pH_24_ or ultimate pH—pH_u_) post mortem with a puncture electrode. Raman spectra were recorded between 60 and 120 min post mortem with a handheld device developed for the conditions prevailing in abattoirs. PLS-R with cross validation (random blocks method) was used to create a multivariate model for the prediction of pH in pork. Figure 2 shows the pH reference values plotted against the predicted pH values from the cross validated PLS-R models for pH_45_ (Figure 2a) as well as for pH_24_ (Figure 2b). Despite a sample set of 96 pork samples, no samples have been separated for a validation check on the performance of the PLS-R models. The figures of merit for the calibration look quite promising for both pH_45_ and pH_24_ with RMSEC = 0.11 and 0.06 pH units, and $R_{\mathrm{CAL}}^{2}$ = 0.82 and 0.85, respectively. The normalized error for the calibration (NRMSEC; see Equation (1)) is below 10% for both pH measurement times.

Expectedly, the figures of merit look somewhat worse for the cross validation with RMSECV = 0.17 and 0.09 pH units, and $R_{\mathrm{CV}}^{2}$ = 0.65 and 0.68 for pH_45_ and pH_24_, respectively. An approximate classification of the obtained $R_{\mathrm{CV}}^{2}$ values by Scheier et al. (2014) [56] can be made according to Ref. [64]. Thus, the reported coefficients of determination from the cross validation can be considered as suited for screening and “approximate” calibrations. Nevertheless, Scheier et al. (2014) [56] reported promising cross validation errors with NRMSECV values between approximately 10% and 12% although they used up to 8 latent variables (LVs) in their PLS-R models. The use of this number of LVs is certainly justifiable with regard to the complexity of the present meat matrix and the target value, but still a comment on the model loadings would have been interesting.

The study of Scheier et al. (2015) [57] aimed at measuring (*inter alia*) pH and recording Raman spectra with a handheld spectrometer of pork at an early post mortem stage (25–40 min post mortem, labled as pH_35_) and 24 h (pH_24_) post mortem. For the first time of measurement, pH and Raman measurements were conducted at the slaughter line in the abattoir, the second one was performed in the laboratory. This work consisted of 151 meat samples from 48 slaughter batches. The calibration in the PLS-R model for the early post mortem stage (pH_35_) yielded a coefficient of determination of $R_{\mathrm{CAL}}^{2}$ = 0.75, however, after cross validation the R^2^ dropped to $R_{\mathrm{CV}}^{2}$ = 0.55. As a consequence, this means that 55% of the variance in the pH reference measurements can be accounted for by the variance in the Raman spectra, which seems rather low if valid conclusions are to be drawn. In the case of pH_24_, the coefficient of variation after cross validation is even lower with $R_{\mathrm{CV}}^{2}$ = 0.31. The reported error of cross validation for pH_35_ is RMSECV = 0.09 pH units in a range of 6.09–6.94 pH units, resulting in a normalized error of NRMSECV = 10% (see Equation (1)). The PLS-R model for pH_24_ yielded an RMSECV = 0.05 pH units in a range of 5.30–5.65 pH units, and thus an NRMSECV of approximately 14%. This cross validation errors again look promising, but they need to be verified with independent validation samples. Considering the fact that 151 meat samples from 48 slaughter batches were available, it is not clear why no samples were kept out of the calibration and cross validation precudure in order to test the performance of the PLS-R models. As long as no independent validation set has been predicted with acceptable RMSEP values, no principal feasability of handheld Raman spectrometers to predict pH of meat should be concluded.

Fowler et al. (2015b) [51] conducted two separate experiments in order to predict (among other meat quality traits) the pH of lamb 24 h post mortem (pH_24_) and 5 days post mortem (ultimate pH, pH_u_) with Raman spectra obtained from a handheld device. Each of the two experiments consisted of 80 randomly selected lamb samples and the measurements were distributed over four consecutive days for experiment 1 and two consecutive days for experiment 2. PLS-R with k-fold cross validation was used to investigate potential correlations of Raman spectra with pH reference values. Although pH was measured with a pH electrode at 24 h and 5 d post mortem, the results for the prediction of pH_u_ (5 d post mortem) using Raman spectra collected at 5 d post mortem were not reported for experiment 1. Instead, Raman spectra recorded at 24 h post mortem were used to predict both pH_24_ and pH_u_. As a result, coefficients of determination of $R_{\mathrm{CV}}^{2}$ = 0.48 and 0.59 were obtained for the prediction of pH_24_ respectively pH_u_ with Raman spectra recorded at 24 h post mortem. According to Ref. [64], these R^2^ values can be considered as acceptable for rough screening. The reported cross validation errors are RMSECV = 0.12 and 0.07 pH units for the prediction of pH_24_ and pH_u_, respectively. Normalization of these errors according to the range of calibration using Equation (1) yields an NRMSECV = 17% for pH_24_ and NRMSECV = 10% for pH_u_. The errors look quite promising, but taking into consideration that these are cross validated errors and that, despite the availability of 80 meat samples, no validation set has been used to evaluate the true performance of the PLS-R models, the reported errors must be treated with caution. This conclusion is further supported by the fact that no reasonable correlation between pH reference values and Raman spectra was obtained in experiment 2 as reported by the authors. Another study of Fowler et al. (2018) [52] with 45 beef loins showed a very weak correlation of Raman spectra with pH reference values at 72 h post mortem between measured and predicted pH ($\rho_{\mathrm{CAL}}$ = 0.12 and $\rho_{\mathrm{CV}}$ = 0.09). The reported cross validation error from a PLS-R analysis is RMSECV = 0.87 pH units, which is even higher than the pH range used for the calibration (pH_CAL-range_ = 5.5–6.1) and yields an NRMSECV = 145%.

Nache et al. (2016) [58] used 96 pork samples for their study of a potential feasability of predicting pH_45_ and pH_24_ from Raman spectra recorded with a handheld device. Also, they investigated cross-predictions, i.e., prediction of pH_24_ from Raman data at 45 min post mortem and vice versa. The authors employed the metaheuristic approach ant colony optimization (ACO) [65] for selecting the best spectral regions in combination with SIMPLS regression [66]. Furthermore, Nache et al. (2016) [58] performed the multivariate data analysis in two ways: on the one hand, they used all 96 samples in a k-fold cross validation procedure and on the other hand, the authors split the data set into calibration and validation samples at a ratio of 70:30. Unfortunately, no ranges for the pH reference measurements at 45 min and 24 h post mortem were reported. Thus, in order to get an idea of the pH calibration range, the study of Scheier et al. (2014) [56] was consulted. Scheier et al. (2014) [56] used the same pork muscle (*m. semimembranosus*) and had the same amount of samples. In their work they reported a range of pH = 5.41–6.8 and 5.28–6.13 for pH_45_ and pH_24_, respectively. Considering this range, the estimated percentage error for pH_45_ is approximately 13% (see Equation (1)) for the cross validation approach (NRMSECV) as well as the validation set approach (NRMSEP). Due to different pre-treatments, the error for pH_24_ for the cross validation approach is estimated beween NRMSECV = 12%–14%. The validation set approach for pH_24_ yields an NRMSEP = 16%. It is important to emphasize, that Nache et al. (2016) [58] tested the performance of their multivariate calibration model with a separate independent validation set and thus the reported error for the prediction of pH_45_ and pH_24_ using Raman spectra may be assessed as realistic with regard to the complexity of the present sample matrix. Unfortunately, only coefficients of determination for the cross validation approach were reported and none for the validation set approach. Nevertheless, R^2^ values between 0.85 and 0.90 for the cross validation approach show the potential of ACO in selecting the most useful spectral areas. The reported figures of merit for the cross-predictions of both pH values were similar to those of the regular predictions.

### 2.3. Prediction of L* Values and Drip Loss with Raman Spectroscopy

A potential correlation of L* values (lightness values) and drip loss with Raman spectra of different meats was investigated in studies of Scheier et al. (2014, 2015) [56,57] and Fowler et al. (2015b, 2018) [51,52]. Furthermore, Scheier et al. (2014) [56] and Fowler et al. (2015b, 2018) [51,52] investigated the possibility to predict a* and b* values using Raman spectroscopy. However, since these attempts were similarly successful as the ones for L* values [Scheier et al. (2014) [56]] or did not work at all [Fowler et al. (2015b, 2018) [51,52]], they will not be further discussed in this review. Concerning the L* values, none of the herein reviewed articles described any restrictions regarding the positioning of the colorimeter on the meat samples, i.e., if particularly positions containing predominantly lean tissue were chosen or if it was taken care to avoid fat tissue. All these studies employed a handheld Raman spectrometer and reported deviating results. Scheier et al. (2014) [56] created a PLS-R model with 96 pork samples for the prediction of L* values at 24 h post mortem from Raman spectra recorded in a cooling room of an abattoir at 60–120 min post mortem. The calibration and cross validation were performed with reference values from a portable colorimeter with a range of L* = 41.0–55.1. The reported errors are RMSEC = 0.7 and RMSECV = 1.9 with $R_{\mathrm{CAL}}^{2}$ = 0.95 and $R_{\mathrm{CV}}^{2}$ = 0.64. Due to circumstances, only 81 of originally 96 pork samples were used for the determination of drip loss. To this end, samples standardized in size were stored at 4 °C over a period of 48 h and drip loss was determined by the difference between initial and final weight. The drip loss ranged from 0.7–9.2%. The prediction of drip loss with Raman spectra measured at 60–120 min post mortem yielded an error of RMSEC = 0.6% with $R_{\mathrm{CAL}}^{2}$ = 0.9 and RMSECV = 1.0% with $R_{\mathrm{CV}}^{2}$ = 0.73. It is quite difficult to assess the true predictive power of a PLS-R model with only calibration and cross validation data available. On the one hand, the RMSEC and $R_{\mathrm{CAL}}^{2}$ look very promising for the prediction of L*, but on the other hand, the error has more than doubled and a remarkable drop of the coefficient of determination is recorded after cross validation. An increase of the percentage error from NRMSEC = 5% to NRMSECV ≥ 13% (Equation (1)) for L* makes it difficult to estimate a test set validated error. As a consequence, no final conclusions on the expected error in a potential implementation in routine analysis can be derived. Furthermore, an $R_{\mathrm{CV}}^{2}$ of 0.64 for L* can be considered as applicable for rough screening [64], which is by no means sufficient enough for analysis in routine operations in the food sector. The cross validation results for the prediction of drip loss can be categorized as good enough for screening and some other “approximate” calibrations [64], but still no reliable statement on the true predictive power of drip loss using Raman spectroscopy can be made without independent validation samples.

The study of Scheier et al. (2015) [57] investigated whether it is possible to predict L* values and drip loss of meat with Raman spectra recorded with a handheld device at an early post mortem stage. For that, Raman spectra of 151 pork samples were recorded 30–60 min post mortem at the slaughter line and L* values of 96 samples were measured 24 h post mortem. The drip loss was determined by the weight difference of 136 pork samples after being suspended in a plastic container for 48 h (from 24 h to 72 h post mortem). For detailed information on the varying sample number, the interested reader is referred to the authors’ original study. PLS-R was used to evaluate the correlation between spectral data and the two target values L* and drip loss. Scheier et al. (2015) [57] reported an NRMSECV of more than 17% (see Equation (1)) for L*, which is rather high considering that this error originates from cross validation. However, due to the very poor coefficient of correlation (R^2^≤ 0.1), the authors did not consider this PLS-R model as having any predictive power. In case of the drip loss, the authors reported an error of RMSEC = 0.4% and RMSECV = 0.6% with $R_{\mathrm{CAL}}^{2}$ = 0.83 and $R_{\mathrm{CV}}^{2}$ = 0.52. These errors look quite low, but taking the range of the reference measurements into consideration, the NRMSECV is somewhat higher than 14% (see Equation (1)). Additionally, the significant drop of the coefficient of determination from calibration to cross validation has to be noted. Accordingly, higher NRMSEP values and lower correlations ($R_{\mathrm{VAL}}^{2}$) are to be expected when an independent validation set is used to test the performance of the drip loss model.

Among other target figures, Fowler et al. (2015b) [51] studied the predictability of L* values from Raman spectra at 24 h and 5 d post mortem using PLS-R. Furthermore, a correlation between drip loss and Raman spectra recorded at 24 h and 5 d post mortem was investigated. This research was conducted on 80 lamb carcases which were measured over four consecutive days. The range of the L* reference values measured with a colorimeter was L* = 36.2–47.7 at 24 h post mortem and L* = 33.8–44.2 at 5 d post mortem. The authors reported an error of RMSECV = 1.96 with 8 LVs and an $R_{\mathrm{CV}}^{2}$ = 0.32. According to Equation (1), this corresponds to an NRMSECV of 17%, which might look promising, but in consideration of the presented coefficient of determination, only a very poor correlation is given between the spectra and the L* reference values. Additionally, Fowler et al. (2015b) [51] used Raman spectra at 24 h post mortem to predict L* values at 5 d post mortem. PLS-R yielded similar results to the previous ones with only 1 LV. Due to the complexity of the meat matrix, it is highly unlikely that the variation in L* reference values is explained in the 1st LV of the PLS-R model. Rather it can be estimated that the model’s explained variance dropped dramatically and thus the model collapsed after the 1st LV. Unfortunately, there are no figures of merit presented concerning the predictability of L* at 5 d post mortem from Raman spectra at 5 d post mortem. Consequently, it might be assumed that these results were inferior and were therefore not reported. The drip loss was determined by comparing the sample weight at 24 h and 5 d post mortem. The samples were stored at −1 °C over this time span. Using Raman spectra at 24 h post mortem for the construction of the PLS-R model, the authors reported a cross validation error of RMSECV = 0.90% in a range of 1.1–6.4% drip loss with an $R_{\mathrm{CV}}^{2}$ = 0.42. Raman spectra at 5 d post mortem yielded an RMSECV of 0.94% with an $R_{\mathrm{CV}}^{2}$ = 0.33. Both cross validation errors correspond to a normalized error of NRMSECV = 17% with regard to the range of the reference values. Concerning the coefficients of determination, it should be pointed out that only 42% and 33% of the variance in the drip loss reference values can be accounted for by the variance in Raman spectra at 24 h and 5 d post mortem, respectively. Taking all this into consideration, the authors’ conclusion that their study demonstrated a certrain potential to predict L* values and drip loss of lamb using Raman spectra recorded with a handheld device is not shared.

In a later study of Fowler et al. (2018) [52] the prediction of L* values and drip loss from Raman spectra with PLS-R did not succeed. In this work the L* values as well as Raman spectra of 45 beef loins were measured at 3 d and 21 d post mortem. The drip loss was determined by weighting the samples after a storage period of 19 d at 3 °C. Although different meats and experimental conditions were used in this study compared to the study of Fowler et al. (2015b) [51], the failure of this work in predicting L* and drip loss from Raman spectra demonstrates that any results concerning these two parameters should be viewed with caution—especially if they are not test set validated.

### 2.4. Meat Spoilage Identification with Raman Spectroscopy

Schmidt et al. (2010) [59] presented a prototype handheld Raman sensor ready for mobile use in meat quality analysis. After testing the wavelength and intensity stability of this prototype, pork samples were investigated with regard to time dependent changes of their Raman spectra. For this purpose, pork samples (number of samples not specified by the authors) were stored at 5 °C in plastic packaging and measured on days 2, 3, 6–17, 20 and 21 post mortem (three weeks) through the packaging and without the packaging. Microbiological reference analysis was performed and expressed in terms of colony-forming units (cfu). Data analysis was conducted using principal component analysis (PCA). The results for both approaches (packaged and unpackaged) are not straightforward, as the authors reported a deterioration of the signal-to-background ratio with progressive storage time, which they attributed to laser induced fluorescence (LIF) coming from porphyrins. This decrease in signal-to-background ratio is mainly responsible for the separation of the time-dependent Raman spectra in principal component 1 (PC 1) of the PCA. Schmidt et al. (2010) [59] assigned PC 2 and PC 3 to changes in protein structure occuring during storage and aromatic amino acids by interpretation of the corresponding PC loadings. Furthermore, a cluster formation along PC 1 of days 2–6 (unspoiled) and 7–9 (started spoilage) can be seen, which is in accordance with the reference analysis, where the threshold of 106 cfu/cm^2^ is reached by days 5–6. Besides this, the authors claimed that PCA was able to indicate the entrance of the steady state of the sigmoid bacterial growth curve obtained from reference analysis at days 9–10. As a consequence, Schmidt et al. (2010) [59] reported that the steady state can be separated from the bacteria’s exponential growth phase until day 9 to 10 with PCA using PC 1 and PC 2. The interpretation of the PCA plot presented in this work is conclusive, although it should be noted that no validation took place and that no classification (in the strict sense, i.e., LDA, SVM-C, projection of unknown samples using a PCA model, etc.) according to the PCA interpretation was conducted. Nevertheless, the authors state that they cannot conclude that the bacteria caused the differences in the Raman spectra leading to the clustering in the PCA. In fact, they claimed to detect the effects of increasing bacterial growth with time. Additionally, Schmidt et al. (2010) [59] performed a PLS-R using the surface concentration of the bacteria on each day and the corresponding Raman spectra. Unfortunately, the authors only reported a coefficient of correlation of R^2^ = 0.969 with 5 LVs, but they did not provide detailed information on how the PLS-R was performed. Thus, no reasonable statement regarding the feasability of predicting bacteria concentration from Raman spectra using PLS-R can be made.

**Figure 3:**
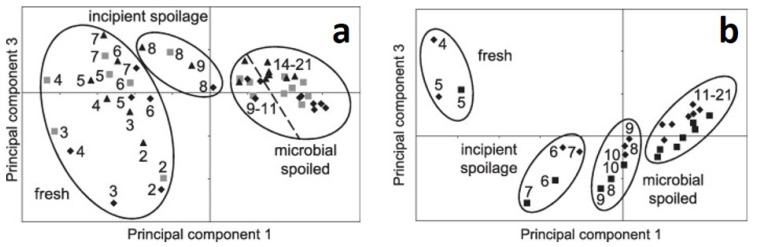
(**a**) PCA scores plot of PC 1 and 3 of porcine LD muscle. (**b**) PCA scores plot of PC 1 and 3 of porcine SM muscle. Stages of bacterial growth are marked with ellipses and the symbols represent the corresponding number of muscles used for each type of muscle. Reprinted from Ref. [60] with permission from Elsevier.

In the study of Sowoidnich et al. (2012) [60] a portable Raman spectrometer was used for fast and non-invasive identification of meat spoilage. The sample set consisted of three porcine *m. longissimus dorsi* (LD) and two porcine *M. semimembranosus* (SM), whereas each muscle was cut into 14 and 16 slices, respectively. All samples were subjected to a storage period of up to three weeks post mortem at 5 °C. Raman measurements as well as microbiological reference analyses (total viable counts—TVC) were conducted daily from day 2 to 21 post mortem. The spectroscopic measurements were analyzed using PCA and an attempt was made to find potential patterns which could subsequently be explained by the results of the reference analyses. The authors presented a PCA scores plot of PC 1 and PC 3 for each investigated muscle (Figure 3a,b). Sowoidnich et al. (2012) [60] stated that the PCA scores and the clustering of samples found therein reflected characteristic stages of the bacterial sigmoid growth curve, i.e., clustering of fresh samples, samples with incipient spoilage and microbial spoiled samples. From examining the PCA loadings of PC 1, the authors identified mainly vibrations of aromatic amino acids and protein conformation sensitive vibrations as being responsible for the separation along PC 1 in both muscles. According to the authors, PC 3 was dominated by signal shifts of tyrosine and phenylalanine bands as well as certain CN and CH vibrations. The interpretation of the separation in PC 1 is conclusive and comprehensible, but the one for PC 3 is not quite complete. The authors’ conclusion, that PC 3 is responsible for changes in the protein structure at an early stage, might be valid for the fresh samples (day 2–7), but the question remains what all the samples arranged along PC 3 have in common which are assigned to different spoilage degrees (see Figure 3a,b). However, it is important to mention that the interpretation of PC 3 is not at all straightforward.

In addition to the experiment described above, the authors studied the effect of meat sterilization using six LD muscles. From this sample set, three muscles were sterilized using a 5% sodium hypochlorite solution, while the other three remained untreated. Each sample was cut into 13 slices, sterile packed and stored under the same conditions as in the previous experiment. Reference analytics and Raman spectroscopic measurements were also conducted daily over a period of 2 weeks post mortem. The authors reported different patterns in the PCA scores plot between sterilized and untreated samples. Similar to the previous experiment, the Raman spectra of the untreated samples revealed a separation according to the reference analyses of fresh samples, samples with incipient spoilage and microbial spoiled samples in PC 1 and PC 2. However, Sowoidnich et al. (2012) [60] reported that such a pattern was not observed in the PCA scores of the Raman spectra of the sterilized samples, since the bacterial surface coverage remained beneath the detection limit for these samples, yet there was a storage-time dependent clustering trend. The authors stated that this separation was not caused by the bacteria, but by the occurance of structural changes that take place in the meat during storage. Due to the fact that no validation samples nor classification methods (see previous paragraph on Sowoidnich et al. (2012) [60]) were used to verify the obtained results, the authors’ final conclusion that their study demonstrated a fast detection of spoiled meat using a portable Raman system is not supported. Rather the presented results only indicate a potential discrimination between fresh and microbially spoiled meat, which requires further profound investigation.

### 2.5. Other Applications of Raman Spectroscopy in Meat Science

*Boar Taint:* The study of Liu et al. (2016) [61] dealt with the detection and classification of boar taint using a handheld Raman spectrometer. Boar taint is an unpleasant odor which occurs in varying intesity when fat or meat of non-castrated male pigs is prepared. This undesired feature is caused by the accumulation of androstenone and skatole in the fat tissue of boars [67]. Currently, tainted meat is commonly detected by trained assessors, but there are also chromatographic [68] and spectroscopic methods [69,70] described in literature. However, the sample set of Liu et al. (2016) [61] consisted of fat tissue from 46 boars including skin, and inner (IL) and outer layer (OL) of subcutaneous fat. The reference analyses were conducted with gas chromatography (GC) and a trained sensory panel, whereby IL and OL were not separated in both cases. For detailed information about the reference analytics, please refer to Liu et al. (2016) [61]. According to this results, the samples were classified into two categories: samples with high boar taint (labeled H; 21 samples) and samples with low boar taint (labeled L; 25 samples), which served as reference values for the multivariable classification. The Raman spectra were recorded seperately on the IL and OL in order to account for any side-dependent effects. Partial least squares discriminant analysis (PLS-DA) was chosen as multivariate classification technique. The authors created five PLS-DA models with distinct input variables based on the spectra obtained on the IL and OL of the sample: only IL spectra, only OL spectra, IL + OL spectra, average apectra of IL and OL and ratio spectra of IL/OL. Liu et al. (2016) [61] reported classification accuracies of 45.5–72.1% samples correctly classified as either H (high tainted) or L (low tainted). However, the highest accuracy was achieved using only IL Raman spectra, while using only OL Raman spectra yielded the lowest accuracy. Furthermore, the authors reported an improvement to more than 80% correctly classified samples using only IL Raman spectra by selecting important wavenumbers according to the model’s regression coefficients. Although the presented results look quite good, it should be kept in mind that the reported classification accuracies are obtained from cross validation. Therefore, independent validation samples are mandatory for conclusions about the model’s true performance in discriminating between samples with high and low boar taint [46]. Nevertheless, the reported cross validated classification accuracies seem promising and further investigations should be conducted. The results might be further improved by application of classification techniques that account for non-linear effects like SVM-C [54]. Apart from that, the authors tried to discriminate between samples which are high in androstenone/high in skatole, high in androstenone/low in skatole, low in androstenone/high in skatole and low in androstenone/low in skatole. Unfortunately, the authors concluded that the accuracy of this PLS-DA classification model was not satisfactory.

*Intramuscular Fat:* Fowler et al. (2015a) [62] investigated the predictability of intramuscular fat (IMF) content and major fatty acid (FA) groups using Raman spectra obtaind from a handheld device. The sample set incorporated 80 lamb carcases which were randomly selected and measured on four consecutive days. Reference analytics for IMF was performed by Soxhlet extraction using an adapted AOAC method [19] and major FA groups were determined by extraction and derivatization according to Ponnampalam et al. [71]. The authors employed PLS-R for the construction of multivariate prediction models for IMF and major FA groups. Considering the range of each determined quantity, all NRMSECV values (see Equation (1)) are in the area of 20%. However, the reported coefficients of determination in a range of $R_{\mathrm{CV}}^{2}$ = 0.01–0.21 for the cross validated PLS-R models are inferior and do not show any signs of correlation between reference values and predicted values from Raman spectra. Therefore, considering the presented figures of merit in the study of Fowler et al. (2015a) [62], the authors’ conclusion that Raman spectroscopy is a promising tool for the prediction of certain major FA groups is incomprehensible. Furthermore, the approach of Raman spectra selection for the prediction of IMF needs to be discussed. The authors subjected the selection of spectra for the chemometric data analysis to two stages: in the first stage, spectra containing any lipid signals were separated from those containing only meat signals and in the second stage, spectra containing only lipid signals and spectra which did not contain exclusively lipid signals were separated from each other. This approach is reasonable for the prediction of FA groups from Raman spectra, since the interest is focussed on lipid tissue only. It is questionable, however, to use only lipid spectra for the prediction of percentage IMF in meat, simply because this procedure produces a lack of relation between lipid tissue and the rest of the meat sample.

## 3. Conclusions

Despite the well known advantages of Raman spectroscopy as being a fast and non-invasive technique which requires only a small amount or no sample pre-treatment at all and the fact that portable handheld devices are available, the dependency on reference analytics is a considerable drawback. For instance, this becomes clear in the case of shear force measurements or the determination of tenderness by a sensory panel in meat science. Especially meat quality traits like the ones just mentioned are affected by modest reproducibility, large variations and subjective perceptions. To a large extent, this is owed to the very complex and inhomogeneous meat matrix, but partly also to the reference methods in general. Therefore, it must be concluded, that the current reference methods must be improved or new methods for reference analysis need to be established. Furthermore, due to the small diameter of the laser spot (according to Ref. [59] approximately 50 µm), only a very small part of the sample is irradiated. Considering the inhomogeneity of the meat matrix, this fact impedes the acquisition of representative Raman spectra.

Unfortunately, the majority of the herein reviewed articles did not utilize independent validation samples in order to test their multivariate calibration models. Subsequently, some authors drew conclusions, which should not be drawn from data that has not been test set validated. Therefore, in order to get an idea of a spectroscopic method’s true performance, reproducibility and robustness, it is essential to use samples, that have never been used before in any calibration model and collect an appropriate number of samples over a long period of time. There might be a certain potential of handheld Raman spectrometers for meat sector-relevant applications, but without the evaluation of the calibration models with independent validation samples, no well-founded statement about the true applicability can be made. This is even more true in view of the meat matrix’ inhomogeneity and complexity.

In the light of the herein reviewed results and depending on the meat producer’s needs, perhaps reflections should take place whether it is expedient to stick with the prediction of individual parameters (e.g., pH, L* value, drip loss, etc.) using handheld Raman spectrometers or to switch to an overall categorization of meat samples into classes of desired and undesired meat quality. In this way, the focus would be on the interaction of all individual meat quality parameters, which *might* lead to more robust and reliable results. First, the meat samples would be classified into desired and undesired meat quality according to the reference analyses. Subsequently, classification techniques like LDA, SIMCA or SVM-C could be used to create classification models, followed by an evaluation using independent validation samples. This would certainly not satisfy all requirements in the meat sector, but might be a valuable support in certain cases. The application of various multivariate approaches on data produced by non-invasive analytical techniques in food science is reviewed in Ref. [72]. 

## Figures and Tables

**Figure 1:**
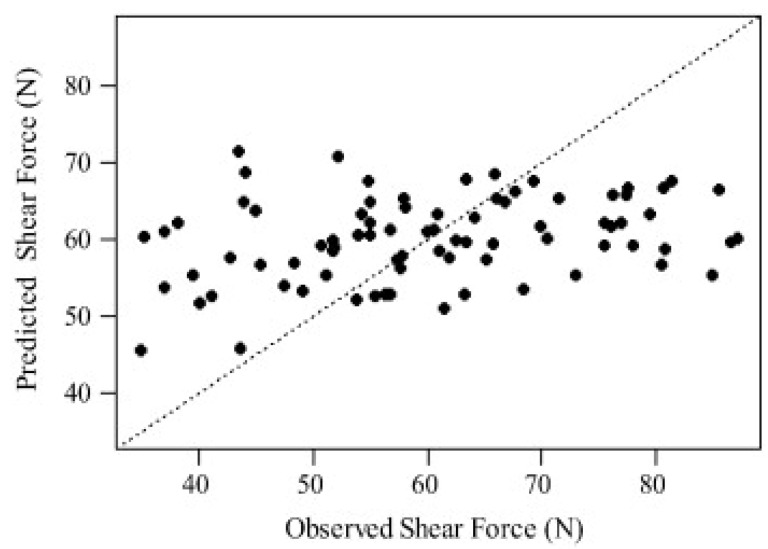
Reference values of shear force against predicted shear force values from Raman spectra using PLS-R and cross validation at 1 day post mortem. Reprinted from Ref. [49] with permission from Elsevier.

**Table 1:** Overview of the target figures discussed in the review article with the corresponding references.

Target Figure	Method	Meat Type	Authors
shear force	PLS-R	lamb	Schmidt et al. (2013) [48]
		lamb	Fowler et al. (2014a) [49]
		lamb	Fowler et al. (2014b) [50]
		lamb	Fowler et al. (2015b) [51]
		beef	Bauer et al. (2016) [53]
		beef	Fowler et al. (2018) [52]
	PLS-DA	beef	Bauer et al. (2016) [53]
tenderness & juiciness	PLS-R	beef	Fowler et al. (2018) [52]
pH	PLS-R	pork	Scheier & Schmidt (2013) [55]
		pork	Scheier et al. (2014) [56]
		pork	Scheier et al. (2015) [57]
		lamb	Fowler et al. (2015b) [51]
	SIMPLS-R	pork	Nache et al. (2016) [58]
	MLR	pork	Schmidt et al. (2013) [48]
	peak intensity ratio	pork	Schmidt et al. (2013) [48]
L*	PLS-R	pork	Scheier et al. (2014) [56]
		pork	Scheier et al. (2015) [57]
		lamb	Fowler et al. (2015b) [51]
		beef	Fowler et al. (2018) [52]
drip loss	PLS-R	pork	Scheier et al. (2014) [56]
		pork	Scheier et al. (2015) [57]
		lamb	Fowler et al. (2015b) [51]
		beef	Fowler et al. (2018) [52]
meat spoilage	PCA	pork	Schmidt et al. (2010) [59]
		pork	Sowoidnich et al. (2012) [60]
boar taint	PLS-DA	pork	Liu et al. (2016) [61]
IMF &major FA groups	PLS-R	lamb	Fowler et al. (2015a) [62]

## Abbreviations

The following abbreviations are used in this manuscript:

ATR-MIR	attenuated total reflection mid-infrared
CAL	calibration
cfu	colony-forming units
CV	cross validation
DFD	dark, firm and dry
FA	fatty acid
GC	gas chromatography
IL	inner layer
IMF	intramuscular fat
LD	*m. longissimus dorsi*
LDA	linear discriminant analysis
LIF	laser induced fluorescence
LOO-CV	leave-one-out cross validation
LV	latent variable
MLR	multiple linear regression
NIRS	near-infrared spectroscopy
NMR	nuclear magnetic resonance
NRMSEC	normalized root mean square error of calibration
NRMSECV	normalized root mean square error of cross validation
NRMSEP	normalized root mean square error of prediction
OL	outer layer
PC	principal component
PCA	principal component analysis
PFN	pale, firm and non-exudative
PLS-DA	partial least squares discriminant analysis
PLS-R	partial least squares regression
PSE	pale, soft and exudative
RFN	reddish, firm and non-exudative
RMSEC	root mean square error of calibration
RMSECV	root mean square error of cross validation
RMSEP	root mean square error of prediction
RSE	reddish, soft and exudative
SM	*m. semimembranosus*
SVM-C	support vector machine classification
TVC	total viable count
VAL	validation
Vis	visible
VOCs	volatile organic compounds
WHC	water-holding capacity
WSBF	Warner-Bratzler shear force

## References

[1] Müller A., Steinhart H. (2007). Recent developments in instrumental analysis for food quality. Food Chem..

[2] Damez J.L., Clerjon S. (2011). Recent Advances in Meat Quality Assessment. Handbook of Meat and Meat Processing.

[3] European Commission (2013). Horse Meat—Questions and Answers. https://ec.europa.eu/food/safety/official_controls/food_fraud/horse_meat/q-ans_en.

[4] Premanandh J. (2013). Horse meat scandal—A wake-up call for regulatory authorities. Food Control.

[5] European Parliament (2017). Parliamentary Questions—Brazilian Rotten Meat Scandal. http://www.europarl.europa.eu/sides/getDoc.do?pubRef=-//EP//TEXT+WQ+E-2017-002022+0+DOC+XML+V0//EN.

[6] BBC News (2017). Brazil Meat-Packing Giants ‘Exported Rotten Beef’. https://www.bbc.com/news/world-latin-america-39311336.

[7] The Guardian (2018). Fear of Meat Scandal as Data Shows Hygiene Breaches at over Half UK Plants. https://www.theguardian.com/world/2018/feb/23/fear-of-uk-meat-scandal-as-data-shows-hygiene-breaches-at-most-plants.

[8] Hunt M.R., Garmyn A.J., O’Quinn T.G., Corbin C.H., Legako J.F., Rathmann R.J., Brooks J.C., Miller M.F. (2014). Consumer assessment of beef palatability from four beef muscles from USDA Choice and Select graded carcasses. Meat Sci..

[9] Van Wezemael L., De Smet S., Ueland Ø., Verbeke W. (2014). Relationships between sensory evaluations of beef tenderness, shear force measurements and consumer characteristics. Meat Sci..

[10] Damez J.L., Clerjon S. (2008). Meat quality assessment using biophysical methods related to meat structure. Meat Sci..

[11] Karumendu L.U., van de Ven R., Kerr M.J., Lanza M., Hopkins D.L. (2009). Particle size analysis of lamb meat: Effect of homogenization speed, comparison with myofibrillar fragmentation index and its relationship with shear force. Meat Sci..

[12] Shackelford S.D., Wheeler T.L., Koohmaraie M. (1995). Relationship between shear force and trained sensory panel tenderness ratings of 10 major muscles from Bos indicus and Bos taurus cattle. J. Anim. Sci..

[13] Warner R.D., Kauffman R.G., Russel R.L. (1993). Quality attributes of major porcine muscles: A comparison with the Longissimus Lumborum. Meat Sci..

[14] Kauffman R.G., Sybesma W., Smulders F.J., Eikelenboom G., Engel B., van Laack R.L., Hoving-Bolink A.H., Sterrenburg P., Nordheim E.V., Walstra P. (1993). The effectiveness of examining early post-mortem musculature to predict ultimate pork quality. Meat Sci..

[15] Van Laack R.L., Kauffman R.G., Sybesma W., Smulders F.J., Eikelenboom G., Pinheiro J.C. (1994). Is colour brightness (L-value) a reliable indicator of water-holding capacity in porcine muscle?. Meat Sci..

[16] Warner R.D., Kauffman R.G., Greaser M.L. (1997). Muscle protein changes post mortem in relation to pork quality traits. Meat Sci..

[17] Honikel K.O., Fischer C. (1977). A rapid method for the detection of PSE and DFD porcine muscles. J. Food Sci..

[18] European Parliament, Council of the European Union (2013). Regulation (EU) No 1308/2013 of the European Parliament and of the Council of 17 December 2013 establishing a common organisation of the markets in agricultural products and repealing Council Regulations (EEC) No 922/72, (EEC) No 234/79, (EC) No 1037/2001 and (EC) No 1234/2007. Off. J. Eur. Union.

[19] AOAC International (1992). Official method 991.36—Fat (crude) in meat and meat products. J. AOAC Int..

[20] Folch J., Lees M., Stanley G.S. (1957). A simple method for the isolation and purification of total lipides from animal tissue. J. Biol. Chem..

[21] Lepage G., Roy C.C. (1986). Direct transesterification of all classes of lipids in a one-step reaction. J. Lipid Res..

[22] O’Fallon J.V., Busboom J.R., Nelson M.L., Gaskins C.T. (2007). A direct method for fatty acid methyl ester synthesis: Application to wet meat tissues, oils, and feedstuffs. J. Anim. Sci..

[23] Díaz P., Nieto G., Garrido M.D., Bañón S. (2008). Microbial, physical-chemical and sensory spoilage during the refrigerated storage of cooked pork loin processed by the sous vide method. Meat Sci..

[24] ISO 21527-1:2008 (2008). Microbiology of Food and Animal Feeding Stuffs—Horizontal Method for the Enumeration of Yeasts and Moulds—Part 1: Colony Count Technique in Products with Water Activity Greater than 0.95. www.ios.org.

[25] Mayr D., Margesin R., Klingsbichel E., Hartungen E., Jenewein D., Schinner F., Mark T.D. (2003). Rapid detection of meat spoilage by measuring volatile organic compounds by using proton transfer reaction mass spectrometry. Appl. Environ. Microbiol..

[26] Lovestead T.M., Bruno T.J. (2010). Detection of poultry spoilage markers from headspace analysis with cryoadsorption on a short alumina PLOT column. Food Chem..

[27] Blixt Y., Borch E. (1999). Using an electronic nose for determining the spoilage of vacuum-packaged beef. Int. J. Food Microbiol..

[28] Balasubramanian S., Panigrahi S., Logue C.M., Gu H., Marchello M. (2009). Neural networks-integrated metal oxide-based artificial olfactory system for meat spoilage identification. J. Food Eng..

[29] Jia W., Liang G., Wang Y., Wang J. (2018). Electronic Noses as a Powerful Tool for Assessing Meat Quality: A Mini Review. Food Anal. Methods.

[30] Prieto N., Pawluczyk O., Edward M., Dugan R., Aalhus J.L. (2017). A Review of the Principles and Applications of Near-Infrared Spectroscopy to Characterize Meat, Fat, and Meat Products. Appl. Spectrosc..

[31] Huang H., Yu H., Xu H., Ying Y. (2008). Near infrared spectroscopy for on/in-line monitoring of quality in foods and beverages: A review. J. Food Eng..

[32] Prieto N., Roehe R., Lavín P., Batten G., Andrés S. (2009). Application of near infrared reflectance spectroscopy to predict meat and meat products quality: A review. Meat Sci..

[33] Schmutzler M., Beganovic A., Böhler G., Huck C.W. (2015). Methods for detection of pork adulteration in veal product based on FT-NIR spectroscopy for laboratory, industrial and on-site analysis. Food Control.

[34] Ellis D.I., Broadhurst D., Clarke S.J., Goodacre R. (2005). Rapid identification of closely related muscle foods by vibrational spectroscopy and machine learning. Analyst.

[35] Ammor M.S., Argyri A., Nychas G.J.E. (2009). Rapid monitoring of the spoilage of minced beef stored under conventionally and active packaging conditions using Fourier transform infrared spectroscopy in tandem with chemometrics. Meat Sci..

[36] Alexandrakis D., Downey G., Scannell A.G.M. (2012). Rapid Non-destructive Detection of Spoilage of Intact Chicken Breast Muscle Using Near-infrared and Fourier Transform Mid-infrared Spectroscopy and Multivariate Statistics. Food Bioprocess Technol..

[37] Cozzolino D., Murray I. (2004). Identification of animal meat muscles by visible and near infrared reflectance spectroscopy. LWT Food Sci. Technol..

[38] Balage J.M., da Luz e Silva S., Gomide C.A., Bonin M.d.N., Figueira A.C. (2015). Predicting pork quality using Vis/NIR spectroscopy. Meat Sci..

[39] Andrés S., Murray I., Navajas E.A., Fisher A.V., Lambe N.R., Bünger L. (2007). Prediction of sensory characteristics of lamb meat samples by near infrared reflectance spectroscopy. Meat Sci..

[40] Renou J., Bielicki G., Bonny J., Donnat J., Foucat L. (2003). Assessment of meat quality by NMR. Spec. Publ. R. Soc. Chem..

[41] Bertram H.C., Ersen H.J. (2004). Applications of NMR in Meat Science. Annu. Rep. NMR Spectrosc..

[42] Straadt I.K., Aaslyng M.D., Bertram H.C. (2011). Assessment of meat quality by NMR-an investigation of pork products originating from different breeds. Magn. Reson. Chem..

[43] Hassing S., Jernshøj K. (2014). Benefits and challenges in applying Raman spectroscopy. Agro FOOD Ind. Hi Tech.

[44] Herrero A.M. (2008). Raman spectroscopy a promising technique for quality assessment of meat and fish: A review. Food Chem..

[45] Ozaki Y., Šašić S., Šašić S. (2008). Introduction to Raman Spectroscopy. Pharmaceutical Applications of Raman Spectroscopy.

[46] Esbensen K.H., Guyot D., Westad F., Houmoller L.P. (2009). Multivariate Data Analysis: In Practice: An Introduction to Multivariate Data Analysis and Experimental Design.

[47] Næs T., Isaksson T., Fearn T., Davies T. (2004). A User Friendly Guide to Multivariate Calibration And Classification.

[48] Schmidt H., Scheier R., Hopkins D.L. (2013). Preliminary investigation on the relationship of Raman spectra of sheep meat with shear force and cooking loss. Meat Sci..

[49] Fowler S.M., Schmidt H., van de Ven R., Wynn P., Hopkins D.L. (2014). Raman spectroscopy compared against traditional predictors of shear force in lamb *m. longissimus* lumborum. Meat Sci..

[50] Fowler S.M., Schmidt H., Van de Ven R., Wynn P., Hopkins D.L. (2014). Predicting tenderness of fresh ovine semimembranosus using Raman spectroscopy. Meat Sci..

[51] Fowler S.M., Schmidt H., van de Ven R., Wynn P., Hopkins D.L. (2015). Predicting meat quality traits of ovine *m. semimembranosus*, both fresh and following freezing and thawing, using a hand held Raman spectroscopic device. Meat Sci..

[52] Fowler S.M., Schmidt H., van de Ven R., Hopkins D.L. (2018). Preliminary investigation of the use of Raman spectroscopy to predict meat and eating quality traits of beef loins. Meat Sci..

[53] Bauer A., Scheier R., Eberle T., Schmidt H. (2016). Assessment of tenderness of aged bovine gluteus medius muscles using Raman spectroscopy. Meat Sci..

[54] Brereton R.G., Lloyd G.R. (2010). Support Vector Machines for classification and regression. Analyst.

[55] Scheier R., Schmidt H. (2013). Measurement of the pH value in pork meat early postmortem by Raman spectroscopy. Appl. Phys. B Lasers Opt..

[56] Scheier R., Bauer A., Schmidt H. (2014). Early Postmortem Prediction of Meat Quality Traits of Porcine Semimembranosus Muscles Using a Portable Raman System. Food Bioprocess Technol..

[57] Scheier R., Scheeder M., Schmidt H. (2015). Prediction of pork quality at the slaughter line using a portable Raman device. Meat Sci..

[58] Nache M., Hinrichs J., Scheier R., Schmidt H., Hitzmann B. (2016). Prediction of the pH as indicator of porcine meat quality using Raman spectroscopy and metaheuristics. Chemom. Intell. Lab. Syst..

[59] Schmidt H., Sowoidnich K., Kronfeldt H.D. (2010). A prototype hand-held raman sensor for the in situ characterization of meat quality. App. Spectrosc..

[60] Sowoidnich K., Schmidt H., Kronfeldt H.D., Schwägele F. (2012). A portable 671 nm Raman sensor system for rapid meat spoilage identification. Vib. Spectrosc..

[61] Liu X., Schmidt H., Mörlein D. (2016). Feasibility of boar taint classification using a portable Raman device. Meat Sci..

[62] Fowler S.M., Ponnampalam E.N., Schmidt H., Wynn P., Hopkins D.L. (2015). Prediction of intramuscular fat content and major fatty acid groups of lamb M. longissimus lumborum using Raman spectroscopy. Meat Sci..

[63] Dutson T.R. (1983). The Measurement of pH in Muscle and its Importance to Meat Quality. Reciprocal Meat Conference Proceeding.

[64] Williams P.C., Williams P., Norris K. (2001). Implementation of Near-Infrared Technology. Near-Infrared Technology in the Agricultural and Food Industries.

[65] Allegrini F., Olivieri A.C. (2011). A new and efficient variable selection algorithm based on ant colony optimization. Applications to near infrared spectroscopy/partial least-squares analysis. Anal. Chim. Acta.

[66] De Jong S. (1993). SIMPLS: An alternative approach squares regression to partial least. Chemom. Intell. Lab. Syst..

[67] Bonneau M., Le Denmat M., Vaudelet J.C., Veloso Nunes J.R., Mortensen A.B., Mortensen H.P. (1992). Contributions of fat androstenone and skatole to boar taint: I. Sensory attributes of fat and pork meat. Livest. Prod. Sci..

[68] Sørensen K.M., Engelsen S.B. (2014). Measurement of boar taint in porcine fat using a high-throughput gas chromatography-mass spectrometry protocol. J. Agric. Food Chem..

[69] Erdmann B., Erdmann B. (2016). Verfahren und Vorrichtung zum Erkennen und Aussortieren von GeruchsauffäLligen Geschlachteten Ebern in Einer Schlachtlinie. Germany Patent.

[70] Sørensen K.M., Westley C., Goodacre R., Engelsen S.B. (2015). Simultaneous quantification of the boar-taint compounds skatole and androstenone by surface-enhanced Raman scattering (SERS) and multivariate data analysis. Anal. Bioanal. Chem..

[71] Ponnampalam E.N., Butler K.L., Pearce K.M., Mortimer S.I., Pethick D.W., Ball A.J., Hopkins D.L. (2014). Sources of variation of health claimable long chain omega-3 fatty acids in meat from Australian lamb slaughtered at similar weights. Meat Sci..

[72] Ropodi A.I., Panagou E.Z., Nychas G.J.E. (2016). Data mining derived from food analyses using non-invasive/ non-destructive analytical techniques; determination of food authenticity, quality & safety in tandem with computer science disciplines. Trends Food Sci. Technol..

